# Ozone-Induced Oxidative Stress, Neutrophilic Airway Inflammation, and Glucocorticoid Resistance in Asthma

**DOI:** 10.3389/fimmu.2021.631092

**Published:** 2021-02-26

**Authors:** Chioma Enweasor, Cameron H. Flayer, Angela Haczku

**Affiliations:** ^1^ UC Davis Lung Center, University of California, Davis, CA, United States; ^2^ Center for Immunology and Inflammatory Diseases, Division of Rheumatology, Allergy and Immunology, Massachusetts General Hospital, Harvard Medical School, Boston, MA, United States

**Keywords:** asthma, oxidative stress, air pollution, ozone, glucocorticoid resistance, IL-17A, neutrophils

## Abstract

Despite recent advances in using biologicals that target Th2 pathways, glucocorticoids form the mainstay of asthma treatment. Asthma morbidity and mortality remain high due to the wide variability of treatment responsiveness and complex clinical phenotypes driven by distinct underlying mechanisms. Emerging evidence suggests that inhalation of the toxic air pollutant, ozone, worsens asthma by impairing glucocorticoid responsiveness. This review discusses the role of oxidative stress in glucocorticoid resistance in asthma. The underlying mechanisms point to a central role of oxidative stress pathways. The primary data source for this review consisted of peer-reviewed publications on the impact of ozone on airway inflammation and glucocorticoid responsiveness indexed in PubMed. Our main search strategy focused on cross-referencing “asthma and glucocorticoid resistance” against “ozone, oxidative stress, alarmins, innate lymphoid, NK and γδ T cells, dendritic cells and alveolar type II epithelial cells, glucocorticoid receptor and transcription factors”. Recent work was placed in the context from articles in the last 10 years and older seminal research papers and comprehensive reviews. We excluded papers that did not focus on respiratory injury in the setting of oxidative stress. The pathways discussed here have however wide clinical implications to pathologies associated with inflammation and oxidative stress and in which glucocorticoid treatment is essential.

## Introduction: Asthma Phenotypes, Glucocorticoid Resistance, and Oxidative Stress

Asthma is a highly heterogenous disease that can be classified into subsets by a number of different categories. Establishment of the appropriate subsets determines treatment approaches (1, 2). According to severity, asthma has mild, moderate, and severe forms (3–5). Asthma severity worsens during exacerbations associated with oxidative stress, the most common causes of which are viral respiratory infections and indoor/outdoor air pollution, including exposure to O_3_ (6–11). Severe asthma is often more difficult to treat than the moderate or mild form of the disease (12, 13).

According to the predominant inflammatory cell type in the airways, asthma can be classified as eosinophilic, neutrophilic, mixed, or paucigranulocytic (3–5). Airway epithelial damage leads to oxidative stress, release of pro-inflammatory mediators and influx of both eosinophils and neutrophils (10). Neutrophils are the predominant inflammatory cells in severe asthma exacerbations (2, 5, 14). These cells are poorly controlled by glucocorticoids (15). Whether a causative allergen can be identified, asthma is also categorized as either allergic or non-allergic (16). Allergic (atopic) asthma is characterized by increased levels of IgE, eosinophilia, exhaled nitric oxide (NO), and Th2-type cytokines (16). Such “Th2 high” asthma can generally be treated with glucocorticoids and biologicals targeting the Th2 cytokine pathways (17). Approximately half of asthmatics however suffer from “Th2 low” asthma in which these pathogenic features cannot be identified. Thus, although Th2 low asthma patients are often resistant to corticosteroids, they cannot benefit from biologic treatment targeting the Th2 pathway either (18, 19). Especially in Th2 low asthma, corticosteroid resistance (the inability to increase FEV1 by 15% after a 7-day course of oral corticosteroids at 20 mg/day) (20) remains a significant clinical problem that continues to increase asthma morbidity and mortality (21–23).

The underlying molecular pathways of glucocorticoid resistant asthma are complex and generally associated with impaired expression and function of the glucocorticoid receptor (GR). GR-α, the classical glucocorticoid receptor isoform (24–27) has a dominant-negative inhibitor, GR‐β, that does not bind corticosteroids. Overexpression of GR‐β is due to abnormal activation of proinflammatory signaling pathways with emerging evidence for a contribution of oxidative stress (8, 10, 28–31). Oxidative stress is defined as an imbalance between reactive oxygen species and the capability of the biological system to detoxify the reactive intermediates or to repair the damage caused by oxidative free radicals (32, 33).

The common causes of oxidative stress potentially linked to glucocorticoid resistance in asthma are summarized in **Table 1**. Amongst the environmental causes our review is focused on inhalational exposure to the toxic air pollutant, ozone (O_3_) as it was found to be a significant contributor to respiratory illness. Specifically, O_3_ induces airway hyperreactivity in mouse models of asthma (6, 86, 87, 89, 91, 98–107), in Th2 low asthma in rhesus macaques (94, 108) and in healthy human subjects and patients with asthma and COPD (6, 7, 59, 109–116). Ground-level (tropospheric) O_3_ is generated by the action of sunlight’s UV rays from precursors (mostly air pollutants containing hydrocarbons, volatile organic compounds [VOC] and nitrogen oxides emitted during fossil fuel combustion). In cities with high O_3_ levels people had an over 30% increased risk of dying from lung disease (117) and children playing outdoor sports had a three times greater chance of developing asthma (118, 119).

**Table 1:** Common causes of oxidative stress linked to glucocorticoid resistance in asthma.

**Environmental exposures**
⚬Allergen exposure (8, 34, 35)
⚬Infections
▪bacterial (36–39)
▪fungal (34)
▪viral:
•influenza (40–43),
•RSV (44–47)
•Rhinovirus (14, 48–52)
•COVID-19 (53–56)
⚬Inhalation of toxic indoor and outdoor air pollutants (57)
•O_3_ (7, 58–60)
•Diesel exhaust (7)
•Wildfire smoke (59, 61–63)
•Tobacco smoke (30)
•Comorbidities
⚬Obesity (64)
⚬Microbiome dysbiosis (65, 66)
⚬Psychosocial stress (67–72)
⚬Circadian rhythm disturbance (Shift work/jet lag) (73–81)
**Cellular pathologies**
⚬Cancer (82, 83)
⚬Endoplasmic reticulum stress (84)
⚬Mitochondrial dysfunction (85)
**Molecular abnormalities**
⚬NRF2 pathway dysfunction (29, 86, 87)
•Decreased expression and function of antioxidant enzymes (eg: superoxide dismutase and catalase) (31, 35)
⚬Decreased concentration of antioxidant scavenger molecules
•Glutathione (84)
•Surfactant protein A and D (88–91)
⚬Deficiency of nonenzymatic/nutritional antioxidants (92)
•vitamin A, C (92), E, beta-carotene
•selenium
•phytochemicals (66)
•lycopene and lutein, resveratrol, flavonoids (64, 93)
•secoisolariciresinol digluconate (94)
⚬Increased production of reactive oxygen/nitrogen species (eg: ROS, RNS) (31, 35)
•Proinflammatory signaling cascade (NF-kB, AP-1, PI3K) (58, 95–97)

O_3_, ozone; RSV, respiratory syncytial virus; ROS, reactive oxygen species; RNS, reactive nitrogen species; NF-kB, nuclear factor kappa B; AP-1, activator protein 1; PI3K, phosphoinositide 3 kinase.

Against O_3_-induced inflammatory injury, the lung mounts immuno-protective mechanisms such as production of the epithelial-cell derived collectin, surfactant protein D (SP-D) (101). Constitutive expression of this molecule in airway epithelial cells is promoted by glucocorticoid dependent transcription (120–122). O_3_-induced oxidative stress not only destroys the biologically active tertiary molecular structure of SP-D (91, 123–125) but it also diminishes glucocorticoid responsiveness and SP-D expression in airway epithelial cells *in vivo* and *in vitro* (122). Below we discuss the potential significance of O_3_-induced oxidative stress in glucocorticoid responsiveness in asthma.

## O_3_-induced airway inflammation and glucocorticoid resistance

**Figure 1:**
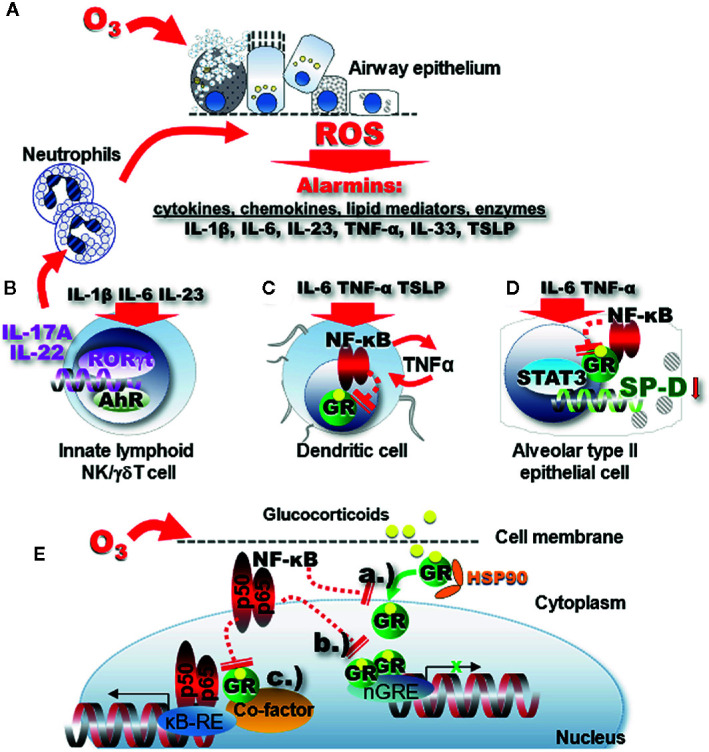
Oxidative stress leads to impaired GR function through proinflammatory signaling. **(A)** Ozone inhalation generates ROS inducing release of alarmins through lipid peroxidation and proinflammatory activation of immune cells in the respiratory mucosal tissue. **(B)** IL-1β, IL-6, and IL-23 activate the RORγt proinflammatory signaling pathway that leads to mRNA activation of the IL-17A and IL-22 genes. The IL-1 family of cytokines and the related IL-33 together with IL-17A and IL-22 induce influx and activation of neutrophils (129, 130). In turn, activated neutrophils in the airway mucosal tissue will release more ROS. **(C)** Proinflammatory signaling activates NF-kB that in turn inhibits expression and function of the GR. Diminished GR function further activates NF-kB forming a vicious proinflammatory cycle. **(D)** The GR uses non-canonical transactivation of the surfactant protein D gene (*sftpd*) through STAT3. GR function impairment in alveolar type II epithelial cells leads to inhibition of the immunoprotective SP-D. **(E)** NF-kB inhibits expression of the GR and interferes with GR function through a.) inhibition of GR nuclear translocation b.) steric hindrance of nGRE binding, and c.) interference with transcription factor “tethering”.

O_3_ exposure results in accumulation of reactive oxygen species (ROS) most likely through lipid peroxidation processes of the pulmonary surfactant phospholipids (60) and cell membranes (126–128). ROS in turn rapidly activate the release of alarmins IL-1β, IL-6, IL-23, IL-33, TNF-α, and TSLP (**Figure 1A**) leading to a cascade of proinflammatory changes in structural and immune cells in the respiratory mucosal tissue (106, 116, 122, 129, 131–136). Activation of the RORγt proinflammatory signaling pathway leads to mRNA transcription of the IL-17A and IL-22 genes (**Figure 1B**) (131, 137–146). The IL-1 family of cytokines together with IL-17A and IL-22 induce influx and activation of neutrophils (129, 130). IL-33 has also been implicated in O_3_ -induced airway inflammation (106, 129, 132–136). IL-33 transcription as well as release is upregulated by O_3_ in the lung in a time dependent manner (106, 134). In the absence of IL-33 or the IL-33 receptor (ST2) acute O_3_-exposure induced epithelial cell injury with protein leak and myeloid cell recruitment and inflammation were enhanced (134). While E-cadherin and zonula occludens 1 and reactive oxygen species expression in neutrophils and airway hyperreactivity were diminished in knockout mice. The enhancement of neutrophil influx was abolished by administration of recombinant IL-33 suggesting a protective role of IL-33 in O_3_-induced epithelial barrier injury in mice.

Activated neutrophils in the airway mucosal tissue will release more ROS. Release of alarmins and influx of inflammatory cells into the airways are the pathological hallmark of severe asthma exacerbations (2, 5, 10, 14). In the healthy lung, the primary inflammatory cells recruited to the airways following O_3_ inhalation are the neutrophilic granulocytes (147, 148). These cells appear in the airways within minutes and accumulate in significant numbers as early as 1–2 h after exposure (89, 106, 149). In healthy human subjects exposed to O_3_ under experimental conditions, a significant airway neutrophilia was associated with a decrease in lung function (7, 147, 150) indicating the pathological significance of these cells. Interestingly, when O_3_ exposure is combined with allergic sensitization in mouse models, asthmatic non-human primates (rhesus macaques) and in allergic human subjects, a marked influx of both eosinophilic and neutrophilic granulocytes is observed (10, 94, 101, 106). While neutrophilia in healthy volunteers could be attenuated by fluticasone propionate (147), studies on mice (107), dogs (151) rhesus macaques (152), and asthma patients (153, 154) showed controversial results on the effectiveness of glucocorticoids in inhibiting O_3_-induced exacerbation of asthmatic airway inflammation. Because asthmatic patients respond to O_3_ with an enhanced airway neutrophilic influx compared with non-asthmatic controls (155), the observation that neutrophils are poorly responsive to glucocorticoids (15) raises a serious concern related to asthma treatment. Indeed, recent studies demonstrated that O_3_ impaired the effects of glucocorticoid treatment in a mouse model of allergen-induced asthma *in vivo* as well as in human cell lines and primary epithelial cells *in vitro* (58, 122, 156). What are the underlying molecular mechanisms of O_3_-induced glucocorticoid resistant neutrophilic airway inflammation in asthma?

## Role of Aryl Hydrocarbon Receptor (AhR) Signaling, IL-17A, and IL-22 in Glucocorticoid Resistant Asthma

The AhR is an intracellular, small molecule ligand-activated transcription factor that regulates gene expression of inflammation-related genes for myeloid and structural cells. AhR is a sensor of xenobiotic chemicals (such as aromatic hydrocarbons) or endogenous indole derivatives [such as kynureine (157)]. AhR mediates environmental signals and is involved in cell differentiation, cell adhesion, mucus and cytokine production (158–160). Upon ligand binding, the AhR complex translocates into the nucleus and heterodimerizes with AhR Nuclear Translocator (ARNT) to induce gene transcription. AhR is an important activator of the genes encoding cytochrome P450 and the cytokines IL-17A and IL-22. The effects of AhR on cell differentiation (including Th17 or Treg polarization) depend on the nature of the ligand and the local cytokine milieu (161, 162).

There are a number of potential mechanisms through which AhR may contribute to glucocorticoid resistance either as a promoter or as an inhibitor. *First*, glucocorticoid responsiveness of airway neutrophilia is regulated by the circadian clock molecule BMAL1 (Brain and Muscle ARNT-Like 1 or aryl hydrocarbon receptor nuclear translocator-like protein 1 [ARNTL]) (163, 164). BMAL1 function is strongly affected by environmental stressors (165) that can be mediated by AhR: Following agonist-induced activation, AhR enters the nucleus, where it can form a heterodimer with BMAL1 impairing its normal transcriptional activities (166) and promoting glucocorticoid resistance. *Second*, AhR interferes with the action of NF-κB, a pro-inflammatory transcription factor and antagonist of glucocorticoid action (see discussion below). For example, NF-κB induces AhR expression, but AhR then regulates NF-κB signaling (159) thereby enabling the glucocorticoid action. *Third*, by interacting with the function of other transcription factors, AhR promotes IL-22 (RORγt), IL-10, and IL-21 (cMaf) as well as aiolos and its own expression (through STAT3). Through aiolos, AhR inhibits expression of IL-2 (159), an inducer of glucocorticoid resistance (167). Thus, on the one hand AhR promotes Th17 cell differentiation, on the other, it induces Th17 cell plasticity into IL-10 producing protective Tr1 cells. While both IL-17A and IL-22 can elicit airway neutrophilia, IL-22 can also play a protective role when produced during epithelial or tissue damage. Recently, chronic ozone exposure induced lung inflammation, airway hyperresponsiveness and tissue remodeling was reported to be associated with increased tryptophan and lipoxin A4 (activators of AhR), and recruitment of IL-17A and IL-22-expressing cells. T cell-specific AhR deletion enhanced lung inflammation indicating that O_3_ exposure activates AhR, to control airway inflammation by reduction of IL-22 expression (168).

IL-17A has been identified as a central player in the pathogenesis of severe asthma exacerbations (169). In human severe asthma patients high levels of IL-17A were found in induced sputum and bronchial biopsies (170). IL-6, the cytokine most prominently induced by O_3_ in the lung (89, 171), and IL-23 (131) directly activate ROR-γt leading to IL-17A expression upon O_3_ inhalation (**Figure 1B**). IL-17A signaling controls neutrophilic airway inflammation (172) mainly through stimulating the release of IL-8 and other pro-neutrophilic factors in the airways (131, 137–146) (**Figure 1B**). The importance of this cytokine in O_3_ exposure-induced exacerbation of allergic airway inflammation was supported in a mouse model in which significant inhibition of IL-17A gene expression by the combined targeting of p38 MAPK activation and oxidative stress was critical in synergistically attenuating airway hyperresponsiveness, eosinophilic and neutrophilic inflammation (107).

IL-17A was also implicated in glucocorticoid resistant asthma (169, 173). For instance, Th17 cells, the main cellular source of this cytokine, were refractory to inhibition with glucocorticoids in asthma, especially, when IL-17A and IL-22 were co-expressed in these cells (169). Increased counts of dual-positive Th2/Th17 cells detected in the BAL fluid of severe asthma patients, were resistant to dexamethasone-induced cell death (169). Glucocorticoid resistance of IL-17 producing cells may be elicited by an elevated expression level of the mitogen-activated protein-extracellular signal-regulated kinase 1 (MEK1) as the MEK-ERK1/2 signaling pathway was shown to interfere with glucocorticoids (174). In a mouse model of airway inflammation, co-administration of dexamethasone with an anti-IL-17A monoclonal antibody significantly inhibited pro-neutrophilic cytokines and the p38 MAPK, NF-kB signaling pathway and reversed O_3_-induced glucocorticoid insensitivity (144).

While Th17 cells were identified as the main producers of IL-17A, O_3_-induced asthma exacerbation in mice did not show T cell activation or migration of T cells into the lung prior to the O_3_- prompted neutrophil influx (106). These results implied that Th17 cells don’t participate in IL-17A release in the early phases of the O_3_-response. Mathews et al. proposed that the source of IL-17A in response to acute O_3_ exposure is the γδ T cell (140). In addition, innate lymphoid cells were shown to be essential and sufficient to elicit development of O_3_-induced neutrophilia (106) and the ensuing airway hyperresponsiveness in mice. These studies suggest the importance of innate immune players in O_3_-induced IL-17A pathways. Interestingly, when compared with Th2 cells ILC2s were found to be relatively steroid resistant in severe asthmatics (51, 156), although they were responsive to steroids in eosinophilic respiratory conditions (175). Increased IL-17A expression was associated with a reduction in GR-α but induced expression of GR‐β in asthmatic airway epithelial cells indicating that the steroid insensitivity in severe asthmatics may be a result of a reciprocal regulation of GR-α and GR-β by IL-17 cytokines. Thus, in addition to Th cells, both IL-17A and IL-22 can be produced by ILC3, γδ T and NK cells, after stimulation with IL-1β, TGF-β, IL-6, or IL-23 and the transcription factor RORγt (168). **Figure 1B** illustrates that IL-17A-mediated neutrophilia in response to oxidative stress feeds back to a vicious cycle by releasing additional ROS into the lung tissue. Further, neutrophils have high constitutive GR‐β expression that may help them resist apoptosis in response to corticosteroid treatment (25). Taken together, oxidative stress-induced IL-17A contributes to glucocorticoid resistance due to an increased activation of phosphokinase signaling pathways, reduction of GR-α, increase of GR-β in IL-17A producing innate immune and T cells thereby promoting neutrophilia.

## Role of the Glucocorticoid Receptor (GR) in O_3_-Induced Glucocorticoid Resistance

Glucocorticoids have significant anti-inflammatory, immunosuppressive and immunomodulatory effects and remain the mainstay of asthma treatment (176). A subset of patients however is refractory to glucocorticoids (12, 177, 178), making their asthma difficult to control (179). Glucocorticoid insensitivity in rare cases, can be a primary genetic trait, but more commonly, it is acquired during inflammatory exacerbations (176). Constitutive GR expression is essential for an adequate glucocorticoid action. Corticosteroid insensitivity can be mediated by decreased function and expression of the GR. Expression of the GR gene (NR3C1) is regulated by complex transcriptional and post translational processes that are modified by airway inflammation (169, 180, 181).

How does the GR work? Glucocorticoids go through the cell membrane and bind to the GR that rearranges the stable GR-heat-shock protein (HSP)90 complex into an activated glucocorticoid-GR complex that translocates to the nucleus (**Figure 1E**). When two of these complexes form homodimers, they bind to specific glucocorticoid response elements (GRE) in the DNA sequence. GRE are located in the promoter regions of glucocorticoid-responsive genes (176). After the recruitment of co-activators or co-repressors, the GR modulates the rate of gene transcription by transactivation or transrepression. Transactivation is triggered by GRE which acts in “trans”, i.e., intermolecularly (this may be considered the opposite of “cis”-acting i.e., intramolecular). On the other hand, transrepression (i.e., inhibition) is the activity of a second transcription factor through protein-protein interaction [reviewed by (182, 183)] (**Figure 1E**). The repressed molecule is usually a transcription factor whose function is to up-regulate gene transcription. Transrepression was first observed in the action of the GR to inhibit the transcriptional promoting activity of the proinflammatory transcription factors AP-1 and NF-κB. Transactivation and transrepression are both important in mediating the anti-inflammatory effects of glucocorticoids. Transactivation GRE up-regulates anti-inflammatory genes such as the NF-κB inhibitor IκBα, the AP-1 inhibitor glucocorticoid-inducible leucine zipper (GILZ) and IL-10. In a mechanism called “tethering” the GR can also interact with other transcription factors (NF-κB, AP-1, signal transducers, and activators of transcription [STAT] or CAAT Enhancer Binding Protein (C/EBP)], and modulate activation of target genes in a monomeric form (184–186). The activated monomeric GR binds to HDAC (histone deacetylase) and interferes with the activation of the κB responsive element (κB-RE) by p65 and p50 heterodimer subunits of NF-κB. Although the main function of HDACs is to modify histones and chromatin structure, HDAC isoforms can have different regulatory functions in the cytoplasm and nucleus. For instance, HDAC1 is considered to be a transcriptional co-activator (187). On the other hand, impairment of HDAC2 function is implicated in corticosteroid resistance of asthmatic and COPD patients (58, 97). Oxidative stress can lead to the reduction of HDAC2 *via* activation of phosphoinositide 3 (PI3K). PI3K induces nitric oxide levels in the asthmatic airways that further hinders the functional ability of HDAC2, as reported in asthmatic smokers (178). Moreover, treatment with theophylline, a medication that restores HDAC2 activity, glucocorticoid sensitivity is also restored (178).

GR expression levels are regulated by transcriptional and post translational mechanisms such as kinase-dependent phosphorylation as well as by homologous ligand down-regulation (by GR agonists) that can be significantly modified by increased NF-κB expression during O_3_-induced oxidative stress (180). Phosphorylation-dephosphorylation is also important in the function of the transcription regulator enzyme, RNA polymerase II. The GR inhibits transcription activation through dephosphorylating RNA polymerase II (188).

Enhanced expression of NF-κB in the nuclear fraction of immune cells paralleled with an impairment of GR nuclear translocation, DNA binding and a decrease in the expression of GR (70). Mutual transrepression has been demonstrated between the GR and NF-κB as well as AP-1. In the highly inflamed airways during oxidative-stress related asthma exacerbation excessive NF-κB and AP-1 activation could be responsible for impaired GR function (27, 176, 189–191). NF-kB not only hinders GR nuclear translocation and directly interferes with GRE-mediated gene transactivation but it can also indirectly “tether” to the GR transcription complex. Importantly, while GR expression is ubiquitous, it is differentially regulated in individual cell types (192). For example cell type-specific increases in NF-κB, in airway epithelial and dendritic cells (**Figures 1C, D**), upon O_3_ inhalation, may significantly inhibit GR expression and modulate allergic airway inflammation [reviewed in (72)].

Glucocorticoid resistance linked to oxidative stress through defective nuclear translocation and GRE binding (**Figure 1E**) (reviewed by Spiers et al. (28, 193, 194). That nuclear translocation of the GR is susceptible to highly pro-oxidative environments was shown by a cultured, fluorescently labeled chimeric GR. Okamoto and colleagues (193) demonstrated that nuclear translocation of GR following acute dexamethasone treatment was impaired in the presence of hydrogen peroxide. This effect was reduced by administration of exogenous antioxidants or by replacing serine for a redox-sensitive cysteine residue. The dissociation of heat shock proteins from the cytosolic GR is also impaired in a pro-oxidative environment, indicating that there may be multiple pathways involved in the cellular response to glucocorticoids (193, 194). Thus, a balanced oxidative state is critical for normal function of the GR.

GR function is also reduced when the molecule is phosphorylated. For example, the proinflammatory signaling molecule, p38MAPK can phosphorylate the GR that blocks nuclear translocation and the ability to bind to DNA leading to decreased ability of the GR to regulate transcription of anti-inflammatory genes (178). Similarly, activation of the MEK-ERK1/2 pathway was shown to antagonize the inhibitory action of glucocorticoids in Th17 cells (174).

Additional mechanisms involve increased expression of GRβ, an isomer of GRα that suppresses the ability of GRα to bind to GRE and induce anti-inflammatory genes. Increase in GRβ is caused by a rise in pro-inflammatory cytokines or through super-antigen such as staphylococcus enterotoxin-induced activation of T lymphocytes (176). Reduced GR expression was reported in asthmatic and COPD patients with insensitivity to corticosteroid treatment (189–191). GR expression can be reduced by homologous ligand down-regulation (upon administration of GR agonists) or other pathways such as transrepression of the GRα isoform by NF-κB in the inflamed tissue (27). It is unclear whether low levels of GR mRNA are due to suppression of promoter activation, decreased mRNA stability, or both (27) during oxidative stress. Importantly, the expression and function of the human GR is distinct from other species. For example, it is unclear whether transrepression of the GRα by NF-κB plays a role in corticosteroid resistance in mice as existence of the dominant negative GRβ isoform [responsible for glucocorticoid resistance (195)] could not be demonstrated in these rodents. Ligand-induced GR down regulation is seen in various tissues and cell types except in T cells (27) suggesting that innate immune and tissue cells may be more susceptible for glucocorticoid resistance. Further studies are still needed to identify the cell types ultimately responsible for mediating the effects of corticosteroid insensitivity in the lung.

## Airway Epithelial Cell Function Is Constitutively Regulated by Endogenous Glucocorticoids

Alveolar type II epithelial cells are the major source of pulmonary surfactant, as well as the immunoprotective lung collectins, surfactant protein (SP)-A and SP-D. SP-D, a glucocorticoid-dependent airway epithelial cell product is critical in the maintenance of pulmonary immune homeostasis (196–203). Individual susceptibility to the effects of O_3_ exposure suggests that inflammatory responsiveness is genetically regulated (204). This is supported by strain dependence of the inflammatory response to O_3_ observed in mice (205–207). A failure of protective immune mechanisms likely plays an important role in shaping the O_3_ effects in the lung. A differential ability of Balb/c and C57BL/6 mice to respond to allergen (208) or O_3_ (89), was inversely proportionate to the amount of SP-D in the lung of these mouse strains (89, 209). Further, when compared to wild-type C57BL/6 mice, the naturally low SP-D producer Balb/c or the SP-D knockout (C67BL/6) animals displayed increased susceptibility *to* and a prolonged recovery period *from* airway inflammation after allergen or O_3_ exposure (89, 210–212).

In addition, O_3_-induced exacerbation of Th2-type airway inflammation in allergen challenged mice was associated with the appearance of abnormal, lower order oligomeric molecular formations of SP-D. Interestingly, in asthmatic rhesus macaques, O_3_ induced de-oligomerization of SP-D was restored by treatment with a flaxseed derivative anti-oxidant (94). Thus, oxidative damage can cause conformational change in the SP-D molecule resulting in a potential loss of its immunoprotective function (91, 213). Glucocorticoids were shown to be necessary for expression of SP-D in epithelial cells (120, 121, 214, 215). Interestingly however, there is no glucocorticoid response elements in the promoter region of the SP-D gene (*sftpd*). This DNA region however contains an evolutionarily conserved STAT3/6 response element in a prominent proximal location. IL-4/IL-13 (activators of STAT6) as well as IL-6 (activator of STAT3) directly upregulated SP-D synthesis in airway epithelial cells *in vitro* and in mice *in vivo* (89, 210). Between SP-D and the STAT3/6-activating IL-6 (89) as well as Th2 cytokines IL-4/IL-13 (216), respectively, negative regulatory feedback mechanisms were identified. In these, inflammatory transcriptional signaling by STAT3/6 would upregulate SP-D synthesis and release. In turn, increased amounts of this protein in the airways would suppress further inflammation through inhibition of proinflammatory cytokine transcription. Lastly, there are indications that STAT3 can be directly phosphorylated by H_2_O_2_ (the molecular product of O_3_ when mixed in water) in airway epithelial cells *in vitro* (217). O_3_ and glucocorticoid treatment had antagonistic effects on SP-D expression and function in the lung, with O_3_ inhibiting glucocorticoid-induced *sftpd* transcription *in vivo* in mice and *in vitro*, in human airway epithelial cell cultures. These results indicated that glucocorticoids sustain vital functions in airway epithelium such as SP-D production, aimed at promoting immune homeostasis. This function is directly perturbed by O_3_-induced oxidative stress.

## Antioxidant Approach for Asthma Treatment

As we discussed, there is a marked role for oxidative stress in asthma, especially in severe exacerbations associated with glucocorticoid resistance. Although this fact has been well established, and according to a WHO estimate, more than 80% of the Earth’s inhabitants used Traditional Medicine/Complementary and Alternative Medicine (TCAM) for their primary healthcare needs (218), a large variety of nutritional, pharmacological, and environmental antioxidant clinical approaches to asthma treatment have been controversial and generally disappointing (33).

Emerging evidence from experimental models shows that successful targeting of oxidative stress in asthma is dependent on activation of NRF2 (Nuclear factor-erythroid 2 related factor 2). NRF2 is an ubiquitous master transcription factor that works through antioxidant response elements (AREs) to induce antioxidant enzyme and cytoprotective protein mRNA expression. Under baseline, “unstressed” conditions, the Kelch-like ECH-associated protein 1 (Keap1) inhibits cellular NRF2 in the cytoplasm and promotes its proteasomal degradation. NRF2 is activated by diverse stimuli such as oxidants, pro-oxidants, antioxidants, and chemopreventive agents (219). NRF2 induces cellular rescue pathways against oxidative injury, abnormal inflammatory and immune responses, apoptosis, and carcinogenesis (219). In a mouse model of asthma Sussan and colleagues used cell-specific activation of NRF2 in club cells of the airway epithelium and found a significantly reduced allergen-induced airway hyperresponsiveness, inflammation, mucus, Th2 cytokine secretion, oxidative stress, and airway leakiness and increased airway levels of tight junction proteins zonula occludens-1 and E-cadherin on the epithelial cell surface. Pharmacological activation of NRF2 during allergen challenge reduced allergic inflammation and airway hyperresponsiveness (220). Administration of the ROS inhibitors, N-acetyl cysteine or apocynin in a mouse model, had no effect on acute injury and lung inflammation but GR-1 antibody depletion of neutrophils significantly reduced ROS production in neutrophils, epithelial cells, interstitial macrophages, and eosinophils (134). In the same study, administration of IL-33 attenuated, while absence of IL-33/ST2 signaling enhanced O_3_-induced airway inflammation and oxidative stress, and diminished zonula occludens-1 and E-cadherin expression highlighting the complex role this cytokine plays during lung injury (134).

In a different study, activation of NRF2 decreased the viability of the wild-type but not of the NRF2-deficient ILC2s resembling the pro-apoptotic effect of glucocorticoids albeit without the involvement of caspase 3-dependent apoptosis or necroptosis. In mice NRF2 activation decreased the number of pulmonary ILC2s and eosinophils suggesting NRF2 activation as a potential alternative strategy for steroid-resistant allergic inflammation (29). Lack of NRF2 in the lung exacerbates oxidative insults including supplemental respiratory therapy (e.g., hyperoxia, mechanical ventilation), cigarette smoke, allergen, virus, bacterial endotoxin and other inflammatory agents (e.g., carrageenin), environmental pollution (e.g., particles, O_3_), and bleomycin (219, 221). Bioinformatic studies elucidated functional AREs and NRF2-directed genes that are critical components of signaling mechanisms in pulmonary protection by NRF2. Association of loss of function with promoter polymorphisms in NRF2 or somatic and epigenetic mutations in KEAP1 and NRF2 has been found in cohorts of patients with acute lung injury/acute respiratory distress syndrome or lung cancer (219).

The role of non-enzymatic antioxidants was studied in a multiple linear regression analysis that revealed significant associations of vitamin C, vitamin E, beta-cryptoxanthin, lutein/zeaxanthin, beta-carotene, and retinol with FEV1% in a large population study (93). Since removal of ROS and RNS from the cells by antioxidants could impair the action of NRF2, one might speculate that antioxidant vitamin administration with simultaneous NRF2 activation could be beneficial in oxidative stress-induced asthma exacerbation, which is a highly proinflammatory condition. To this effect, a dietary flaxseed compound (LGM2605) a synthetic form of the lignan secoisolariciresinol digluconate (SDG) was identified as both an antioxidant and an activator of NRF2. SDG demonstrated strong protective actions against different sources of oxidative damage (222, 223) supporting the potential for antioxidant approaches for asthma treatment. A cohort of asthmatic macaques from the California National Primate Research Center was identified to naturally develop airway hyperresponsiveness (224). These animals display no overt airway inflammation or Th2 cell activation and their peripheral blood mononuclear cells are unresponsive to glucocorticoids (224). Thus, these animals represent “Th2 low” glucocorticoid resistant asthmatic patients and are therefore uniquely poised for investigation of novel alternative or adjuvant approaches to glucocorticoid treatment. A 7-days treatment with LGM2605 of these macaques that received a single exposure to O_3_ or air (as control) not only prevented the O_3_-induced exacerbation of airway hyperresponsiveness but also significantly improved baseline lung function (94). These studies highlight the significance of oxidative stress in the effect of O_3_ on airway hyperresponsiveness and support the idea that anti-oxidant treatment may be beneficial in glucocorticoid resistant, Th2 low asthma.

## Conclusions

Severe glucocorticoid resistant asthma continues to increase morbidity and mortality despite the advent of new powerful biological treatments that target proinflammatory cytokines. Scientific and clinical evidence is emerging that alternative and adjuvant therapeutic approaches could significantly contribute to reducing and/or controlling severe asthmatic symptoms. Harnessing antioxidant mechanisms may have a special importance in this effort as oxidative stress has been clearly demonstrated to worsen steroid resistance in severe asthma. The pathways we discussed here are however widely applicable to clinical conditions associated with inflammation and oxidative stress and in which glucocorticoid treatment is essential. One recent example of this is the wide variability of effectiveness observed by dexamethasone treatment of severe COVID-19 patients (225–227). Our assessment of the literature raised a number of interesting questions that require future clarifications. For example, what is the importance of the different cell types in mediating glucocorticoid resistance in asthma? Does the nature of oxidative stress depend on its etiology? What role do AhR-related mechanisms play and how does transcriptional regulation of the circadian clock figure into glucocorticoid responsiveness? Is it possible to increase expression and the protective function of molecules like SP-D? What are the effects of simultaneous molecular targeting of oxidative stress, inflammation, and NRF2 pathways? How feasible it is to translate experimental data to human studies and ultimately to clinical application? Greater understanding of how oxidative stress affects asthma and steroid resistance may lead to novel therapies that could improve the lives of millions.

## Author Contributions

CF drafted the ozone-related sections. CE drafted the glucocorticoid resistance-related sections. AH revised the draft, edited, and finalized the paper. All authors contributed to the article and approved the submitted version.

## Funding

T32ES007059 (CF) and T32 HL007013 (CF); P30ES023513 Pilot Grant (AH), R21AI116121 (AH), R41AI132012, and 2R42AI132012 (AH); T32HL116275 (CF).

## Conflict of Interest

The authors declare that the research was conducted in the absence of any commercial or financial relationships that could be construed as a potential conflict of interest.
